# ‘Excited Delirium’, acute behavioural disturbance, death and diagnosis

**DOI:** 10.1017/S0033291722001076

**Published:** 2022-07

**Authors:** Terry McGuinness, Maurice Lipsedge

**Affiliations:** 1 UCL Faculty of Laws, London, UK; 2 Emeritus Consultant South London and Maudsley NHS Foundation Trust, Honorary Clinical Senior Lecturer Guy's, King's and St Thomas's School of Medical Education, London, UK

**Keywords:** Acute behavioural disturbance, coroners, emergency psychiatry, excited delirium, restraints

## Abstract

In the 1980s the traditional Hippocratic term excited delirium was transplanted from the bedsides of febrile, agitated and disoriented patients to the streets of Miami. Deaths in custody of young men who were intoxicated with cocaine and who were restrained by the police because of their erratic or violent behaviour were attributed to excited delirium. The blood concentrations of cocaine in these subjects were approximately ten times lower than the lethal level and other factors which might have contributed to the fatal outcome, such as the police use of neck-holds, choke-holds or ‘hog-tying’, were relegated to a minor role compared with the reframed ‘diagnosis’ of excited delirium. Over the course of the next few decades ‘excited delirium’ might be applied to virtually any highly agitated person behaving violently in a public place and who subsequently died in custody while being restrained or shortly afterwards. Expert witnesses, mainly forensic pathologists, testified that the deceased's death was probably inevitable given the perilous nature of excited delirium, even though this diagnostic entity lacked any consistent neuropathological basis and depended entirely on observed behaviour. This history of the rise and fall of this disputed diagnosis is a partial response to the sociologist Phil Brown's 1995 paper asking who benefits, or at least avoids trouble, by the identification and use of a diagnosis.

## Introduction

At the close of the trial of Officer Derek Chauvin for the murder of George Floyd, defence counsel Eric Nelson expounded on the importance of the burden of proof. In his closing submissions he told the jury ‘The state must convince you beyond a reasonable doubt that Mr. Floyd was not experiences [*sic*] excited delirium that contributed to the cause of his death.’ (CNN Newsroom, 2021) Those familiar with judicial enquiries into deaths that followed police use of restraint will not have been surprised to see the defence raise ‘excited delirium’ as a possible explanation for Floyd's death. Over the past four decades it became a diagnosis regularly asserted by those acting for officers, or for the manufacturers of conducted energy weapons (CEW) such as the TASER stun gun, to explain fatal outcomes and to downplay the significance of police officers' use of force.

In this paper, we explain how this previously uncontroversial psychiatric term was radically changed and cited with increasing frequency following deaths in custody and institutional settings. What was once a description of a cluster of symptoms arising from an underlying organic cause, identifiable by clinical and laboratory investigation, now represents a syndrome which defies post-mortem examination but which is said to be so perilous that it renders immaterial the other circumstances of the death. We first describe the typical presentation of someone said to be suffering from excited delirium. We then set out how this diagnostic label came to be distorted in Miami in the early 1980s. Borrowing Ian Hacking's theory of ‘transient mental illness’, we describe the febrile environment that allowed this rebranding to gain traction so quickly. Turning to the dangers of physical restraint, we address the arguments put forward by proponents of excited delirium who have sought to play down the risks involved in restraint. These include the assertion that excited delirium is merely the modern name for the older, nineteenth century condition of Bell's Mania. Starting with the Canadian enquiries into the death of Robert Dziekanski in 2007, which represents the turning point in the excited delirium story, we chart the divergence in attitudes towards excited delirium between Canada and the United States. We then note the greater scrutiny of excited delirium claims in Europe before discussing what future there may be for this increasingly contested diagnosis.

## The typical ‘excited delirium’ case

The term excited delirium is currently used by forensic pathologists, medical examiners and emergency physicians in North America to explain the following constellation of events. There is an unexpected death in custody or in a medical institution during or following restraint. The deceased, usually a male in his 20s or 30s, was restrained after behaving in a violent or uncontrollable manner which had presented a severe threat to his own safety and/ or that of others. His behaviour prior to and during restraint involved extreme physical exertion and manifested profound emotional and psychological disturbance. The deceased may have been intoxicated by cocaine, amphetamines or other psychostimulants, with or without alcohol. Alternatively, he may have had a history of a bipolar or schizophrenia spectrum disorder. The post-mortem examination fails to provide a satisfactory explanation for the cause of the death, which is then attributed, by a process of exclusion, to excited delirium (Lipsedge, 2016).

Many American police forces have embraced the description provided by Michael Curtis MD, who has described someone presenting with these signs as being already on ‘the freight train of death’ before the police arrive on the scene (Curtis, 2007 as cited in Kroll & Ho, 2009, p. 425). He grouped the identifying factors under the mnemonic ‘NOT A CRIME’:

‘N: Patient is **naked** and sweating from hyperthermia

O: Patient exhibits violence against **objects**, especially glass

T: Patient is **tough** and unstoppable, with superhuman strength and insensitivity to pain

A: Onset is **acute** (e.g. witnesses say the patient ‘just snapped!’)

C: Patient is **confused** regarding time, place, purpose and perception

R: Patient is **resistant** and won't follow commands to desist

I: Patient's speech is **incoherent**, often with loud shouting and bizarre content

M: Patient exhibits **mental** health conditions or makes you feel uncomfortable

E: EMS should request **early** backup and rapid transport to the ED [hospital emergency department]’ (Wesley, 2011).

Whilst the US National Association of Medical Examiners and the American College of Emergency Physicians consider excited delirium to be a mental health condition, it is, however, not recognised as such by the American Medical Association, the American Psychiatric Association or the World Health Organisation. Nor is it to be found in the most recent Diagnostic and Statistical Manual of Mental Disorders (DSM V). A diagnostic entity requires a standardised definition, a specific diagnostic test and a unique pathophysiological mechanism with a consistent morbid anatomical basis or a specific aetiology. By contrast, excited delirium has been defined mainly on the basis of subjective descriptions of severely agitated behaviour.

British psychiatrists prefer the term acute behavioural disturbance (ABD) to describe such bouts of alarming, frantic, irrational and potentially dangerous physical activity (Lipsedge, 2016). In their authoritative handbook ‘Psychiatric Intensive Care’, Beer, Pereira and Paton gave a comprehensive descriptive definition of ABD requiring urgent intervention:

It usually manifests with mood, thought or behavioural signs and symptoms and can either be transient, episodic or long-lasting. *It can have either a medical or a psychological aetiology* and may reflect a person's limited capacity to cope with social, domestic or environmental stressors. The use of illicit substances or alcohol can accompany an episode of acute disturbance, or can be causative. The acute disturbance can involve threatening or actual violence towards others, the destruction of property, emotional upset, psychological distress, active self-harming behaviour, verbal abuse, hallucinatory behaviour, disinhibition, disorientated or confused behaviour and extreme physical over-activity – ‘running amok’ (Beer, Pereira, & Paton, 1998). [emphasis added]

ABD is merely a descriptive term that makes no claim to having a unique pathophysiology. Such a presentation can be triggered by a very wide range of physical, psychological and situational factors. As Professor Keith Rix has pointed out (in personal communication with the authors), it has no more diagnostic or predictive value than a pyrexia of unknown origin. This is in stark contrast to the new claims made about excited delirium, said to be fatal in and of itself.

## Miami, cocaine and the distortion of excited delirium

The outbreak of diagnoses of excited delirium can be traced back to the 1981 death of a cocaine-intoxicated Colombian drug smuggler in Miami. His case history was described in the Annals of Emergency Medicine by a psychiatrist called David A Fishbain and a forensic pathologist named Charles V Wetli in a paper entitled ‘Cocaine Intoxication, Delirium and Death in a Body Packer’ (Fishbain & Wetli, 1981). This smuggler had swallowed a number of rubber finger stalls crammed with cocaine. Most of the contraband successfully exited per ano but one ruptured in his digestive tract, releasing a flood of cocaine and causing paranoia and severe agitation. His behaviour became so dangerous that he had to be restrained by six hospital attendants and he died a few hours later. His death was attributed to cocaine intoxication presenting as ‘excited delirium’ (Fishbain & Wetli, 1981). Four years later, the same psychiatrist and pathologist published a study of the deaths of seven recreational cocaine users who were suffering from cocaine-induced psychosis (Wetli & Fishbain, 1985, p. 873). Each of these individuals had also become paranoid and their erratic and violent behaviour led to them being restrained (four of them being ‘hog-tied’). Six of the deaths occurred in police custody. Their average blood cocaine concentration was sub-lethal (Wetli & Fishbain, 1985, p. 878). The deaths were attributed to cocaine intoxication ‘with a psychiatric presentation of excited delirium’ (Wetli & Fishbain, 1985, p. 873). Wetli and Fishbain concluded that ‘police and emergency paramedical personnel should be aware of the potential for sudden death in association with excited delirium’ (Wetli & Fishbain, 1985, p. 879). Wetli's enthusiasm for his new diagnosis bordered on the evangelical, eventually leading to criticism that he was ‘stretching the evidence to fit his theory’ (Garcia-Roberts, 2010):

Wetli used the same term to explain the deaths of 32 Black women occurring in Miami over the 1980s, proposing that a combination of cocaine use and sexual intercourse had led to their demise. Police subsequently attributed the deaths to asphyxiation by a serial killer (Budhu, 2020).

The ‘signs of delirium’ listed by Wetli and Fishbain as those most commonly observed in these recreational cocaine users were fear, panic, shouting, physical violence, hyperactivity, unexpected strength and thrashing, particularly after restraints were applied (Wetli & Fishbain, 1985 p. 874). These authors employed standard medical terminology – cocaine intoxication and cocaine-induced psychosis – to capture both the confusional and the psychotic clinical features. However they needlessly deviated from conventional nosology when they inserted the term ‘excited delirium’. Since the features of the 1980 DSM III diagnostic category of cocaine intoxication included psychomotor agitation, perspiration, fighting, confusion, incoherence, paranoia, bizarre behaviour, impaired judgment and hypervigilance, it might be thought there was no need for a new label for this presentation. Wetli and Fishbain's application of the standard Hippocratic term delirium – a common clinical condition (according to the NICE guideline ‘Delirium: prevention, diagnosis and management’, published 28 July 2010 and last updated 14 March 2019, the prevalence of delirium in people on medical wards in UK hospitals is about 20% to 30%, and 10% to 50% of people having surgery develop delirium; in long-term care the prevalence is under 20%) – to designate a very different clinical picture was not only otiose but a distortion of that established diagnosis. Why invoke delirium when the more specific diagnostic category of psychostimulant intoxication was already well established in the nosological system?

For most physicians, delirium means a fluctuating level of consciousness and a pronounced change in cognition with impaired awareness, attention and concentration, together with the inability to register new information. There are also a reversed sleep/wake pattern and visual hallucinations together with misperceptions, persecutory delusions, incoherent speech and bizarre, disruptive and injurious behaviour. The delirious patient may be either overactive or underactive and there is always, by definition, an underlying medical cause (which might be infective, metabolic, inflammatory, toxic, neoplastic etc). Fishbain and Wetli appropriated the term ‘excited delirium’ from ZJ Lipowski's chapter on organic disorders in Kaplan, Freedman and Sadock's standard three volume Comprehensive Textbook of Psychiatry (Lipowski, 1980). Lipowski deployed the term to describe the overactive sub-category of delirium. He would have had in mind the feverish, confused and fearful elderly patient, who might be picking at the bed clothes or repeatedly trying to get out of his or her sick bed (American Psychiatric Association, 2000, p. 136), not the wild and violent intoxicated young body packer in Miami.

Following the Miami characterisation of people with excited delirium as hyperactive, super-strong and impervious to pain, the misleading use of this term became widespread within the American forensic pathology milieu. Over time, its proponents began to claim that excited delirium explained deaths in custody unrelated to recreational drugs and within twenty years ‘for all practical purposes an acute psychotic episode with agitation and violence [was] synonymous with excited delirium’ (DiMaio & DiMaio, 2006, p. 4). By 2009 a major public enquiry in Canada found the term was being applied even more broadly, to people merely ‘highly agitated and often under the influence of stimulants/drugs or suffering from a mental condition’ (Braidwood, 2009, p. 544).

## Transient mental illness and the ‘ecological niche’ which produced excited delirium

The sudden emergence of novel and disputed diagnostic labels, with concomitant explosions of related research and publication, has intrigued the Canadian philosopher Ian Hacking. He explored the phenomenon in his book Mad Travelers, in which he told the story of fugue – ‘a forgotten epidemic of insanity’ – inaugurated in 1887 by Albert Dadas, a compulsively wandering gas fitter from Bordeaux, and his doctor Philipe Tissié (Hacking, 2002). According to Tissié, those suffering from fugue felt an overwhelming compulsion to travel with no obvious motive and towards no particular destination. The fugueur seemed impervious to the hazards of aimless travel and to the legal, medical and physical risks of wandering. When he surfaced from his automaton-like state, the wanderer had no idea where he had been and why. The epidemic of fugue – or as Hacking prefers, the epidemic of *diagnoses* of fugue (Hacking, 2002, p. 26) – had its epicentre in Bordeaux. There were reports of the condition from as far afield as central Europe but it did not ‘take’ in the United Kingdom or in the United States. It lasted until its rejection at the 1909 Congress of Neurologists and Alienists, held in Nantes.

Hacking saw Tissié's diagnosis of fugue as an example of what he has termed a ‘transient mental illness’. By transient, Hacking did not mean an illness that comes and goes in this or that patient but one that only appears at a certain time and in certain places, ‘for reasons which we can only suppose are connected with the culture of those times and places’ (Hacking, 2001, p. 100), before then fading away. Hacking's other examples of transient mental illness include hysteria in late 19th century France and multiple personality in 1980s America. Hacking proposed that transient mental illnesses emerge from an ‘ecological niche’ created by the convergence of particular social circumstances of the day. He saw the outbreak of diagnosing of fugue as the result of a combination of the burgeoning of popular tourism, the golden age of travel journalism, the desertion of young men from the dull life of the continent's conscripted armies and France's strict anti-vagrancy laws of 1885. These factors led Dr Tissié, who just happened to be an enthusiastic advocate of outdoor exercise who likened the French countryside to an exercise track, to find in Albert Dadas' strange compulsion evidence of a new mental illness. We view excited delirium as a modern example of a transient mental illness.

What then was the ‘ecological niche’ that allowed excited delirium to emerge? We say it was society in south Florida in the early 1980s, an environment created by converging sociocultural influences that included the collapse of community psychiatric facilities, the rise in authoritarian policing and the start of the cocaine epidemic.

President Nixon's withdrawal of federal funding for community mental health centres – set up on President Kennedy's initiative in 1963 to provide high quality care for former long-term mental hospital patients – eventually led to widespread conspicuous vagrancy among discharged psychiatric patients, with a considerable number ending up in prison (Brown, 1985). In this failure of the community mental health effort, Julian Leff located the preconditions for the subsequent ‘rise of a new biologism, a more strictly biomedical and asocial view of mental health and illness’ (Leff, 2010). At the same time, the decisive shift to the right in criminal justice policies under Richard Nixon became established practice under Ronald Reagan. He described the policing of America's cities in military terms, promising to ‘run up the battle flag’ to wage a ‘planned, concerted campaign’ to win the ‘war on drugs’ (Associated Press, 1982). As his law-and-order politics was adopted by Democratic and Republican mayors alike, there was a concomitant rise in allegations of police brutality and misconduct (Felker-Kantor, 2017). The increasingly punitive policies of the next decade were largely the response to the panic caused by the epidemic of crack cocaine.

Throughout the 1970s, cheap cocaine was smuggled in ever-increasing quantities into rural southern Florida through the Bahamian corridor from Central and South America (Farber, 2019 p. 35). It was then collected and brought to Miami for distribution. The cheap and rapidly effective cocaine alkaloid ‘crack’ arrived in Miami no later than 1980 (Farber, 2019, p. 43). Between 1976 and 1985, cocaine-related medical emergencies rose to nine times their previous level and nationally cocaine-related deaths increased elevenfold (Sadock, Sadock, & Ruiz, 2017, p. 1281). Charles Wetli, the local medical examiner who co-inaugurated the excited delirium epidemic, described how cocaine deaths in Dade County – which includes the city of Miami – had increased from one or two each year in the 1970s to nearly two per week by the middle of 1986 (Wetli, 1987). Meanwhile amphetamine use, which had started in the 1960s, peaked over the next 30 years. It was in this environment that the unexplained deaths of highly agitated individuals in police custody began to be ascribed to excited delirium.

## Dangerous restraint techniques

The American Civil Liberties Union and civil rights attorneys in the United States have long argued that a post-mortem diagnosis of excited delirium is used to cover-up police officers' use of dangerous restraint (Garcia-Roberts, 2010; Sullivan, 2007). Their warnings have gone largely unheeded. One recent example from many is the death in December 2018 of Gregory Edwards in Brevard County, Florida. A US Army veteran medic with diagnosed post-traumatic stress disorder, Edwards was arrested following an altercation in a mall parking lot. His wife told the arresting officers she suspected he was experiencing an episode relating to his PTSD. Upon his arrival at the county jail, Edwards fought with correctional officers who responded with knee strikes, punches and the use of both pepper spray and a CEW. Edwards was then strapped into a restraint device nicknamed ‘the Devil's chair’ and a spit hood was placed over his head. He became unresponsive and died the following day in the hospital. The sheriff's office's statement on the death made no reference to the use of force and suggested ‘previous inhalant abuse’ was a possible cause of the ‘medical event’ (Brevard County Sheriff, 2020). The local medical examiner found the cause of death to be excited delirium (Ray, 2020).

Professional and public concern about the use of restraints and deaths in custody predate claims of death by excited delirium. In 1982, three years before the publication of Wetli and Fishbain's second paper, the American Journal of Forensic Medicine and Pathology published a report by two forensic pathologists, Reay and Eisele, that drew attention to the dangers of law enforcement neck holds. As ‘the potential for a fatal outcome is present each time a neck hold is applied’, they recommended the use of this mode of restraint be ‘viewed in the same way as firearms’ (Reay & Eisele, 1982). One warning was particularly prescient. Emphasising how this dangerous restraint technique differs from the use of a firearm in that ‘its fatal consequences can be totally unpredictable’, Reay and Eisele highlighted how its use ‘can turn a simple arrest where the suspect offers resistance to being placed in handcuffs into a death which is publicly scrutinised for potential criminal prosecution of the participating officer’ (Reay & Eisele, 1982). It is precisely this scenario in which excited delirium became the accused officer's defence of choice. Over two decades later, Theresa and Vincent DiMaio, a psychiatric nurse and a forensic pathologist who gave expert evidence on behalf of officers in numerous death investigations, dedicated their influential book ‘Excited Delirium, Cause of Death and Prevention’ ‘to all law enforcement and medical personnel who have been wrongfully accused of misconduct in deaths due to excited delirium syndrome’ (DiMaio & DiMaio, 2006, p. *v*).

In a chapter titled ‘Traditional Explanations for Deaths Due to Excited Delirium Syndrome’ the DiMaios rejected concerns over the dangers of restraint:

One [explanation] is that death is the result of positional or restraint asphyxia, the other that the death results from the use of a neck hold. The authors feel that neither of these propositions explains most deaths associated with excited delirium syndrome.

They cited experiments conducted by Dr Theodore Chan and others in which volunteers were restrained in the hog-tied position (handcuffs applied behind the back and tied to leg or ankle restraints) following a bout of strenuous exercise. Chan's team had found that whilst this restraint resulted in restricted pulmonary functioning, it did not cause clinically relevant changes in ventilation or oxygenation (Chan, Vilke, Neuman, & Clausen, 1997). To address criticism that deaths are due to officers kneeling on the restrained person, Chan et al. later conducted further experiments, now placing weights on the backs of volunteers restrained in the hog-tied position. Their conclusion was that this restraint position, even with weight applied across the person's back, did not cause hypoxia or hypoventilation (Chan, Neuman, Clausen, Eisele, & Vilke, 2004). The DiMaios saw the results of these tests as confirmation of the absence of proof that police restraint by kneeling on individuals compromises their respiration (DiMaio & DiMaio, 2006, p. 37). A critical fault of Chan's work not addressed by the DiMaios is that it is impossible to replicate amongst healthy volunteers in a clinical trial the extreme fear felt by many of those restrained in the streets. The volunteers in the laboratory did not fear those conducting the experiment and knew their safety was assured. The extreme exertion against restraints exhibited by those later said to have died of excited delirium came from a belief that they were in mortal danger. The physiologically dangerous effects of their struggle against restraint, including metabolic acidosis, hyperkalaemia and rhabdomyolysis, were absent in Chan's tests (Lipsedge, 2016, p. 18–19).

The DiMaios were even more dismissive of fears as to neck holds:

If neck holds are inherently dangerous, then deaths should be common in practitioners of judo. Koiwai reported in 1987 that he could not find any deaths due to shime-waza from the inception of judo in 1882 (DiMaio & DiMaio, 2006, p. 40).

These views were far from universal however. US Department of Justice guidance issued to police officers in June 1995 had warned of the dangers of restraining a person in a face-down position (National Institute of Justice, 1995). Its recommendations on preventing deaths included releasing people from the prone position as soon as they are handcuffed, not sitting on them and never hog-tying. North of the border, in a 1998 review of 21 unexpected deaths in custody, Michael Pollanen and colleagues from the Office of the Chief Coroner for Ontario warned that ‘law enforcement officers and others should bear in mind the potential for the unexplained death of people in states of excited delirium who are restrained in the prone position or with a neck hold’ (Pollanen, Chiasson, Cairns, & Young, 1998, p. 1603). They concluded that ‘the possibility that positional asphyxia contributes to unexpected death in people in states of excited delirium cannot be ignored’ (Pollanen et al., 1998, p. 1606). In the United Kingdom, as early as 2004 a Metropolitan Police review recognised that the term excited delirium ought to be removed from its documentation following a coroner's warning that its use ‘encourages failure to recognise the multi-factorial pathophysiology’ of deaths following restraint (Metropolitan Police Service, 2004).

To make their case for excited delirium, the DiMaios cited 22 cases from their own experiences. Most of the individuals in these vivid examples were already medically vulnerable due to underlying cardiovascular problems and/or obesity. To deflect criticism that such cardiovascular vulnerabilities were insufficient to explain these deaths, the DiMaios proposed excited delirium as not only a description of the wild and dangerous behaviour but also as the primary cause of death. Regarding the certification of death, the DiMaios proposed that if there is no autopsy evidence of sufficient trauma to explain death, the cause of death could be ‘signed out’ as excited delirium with the ‘struggle’ and other factors such as cocaine intoxication designated as contributory causes (DiMaio & DiMaio, 2006, p. 80).

## Creating a history for excited delirium

Like other proponents of excited delirium, the DiMaios do not accept that it emerged in the 1980s and seek to tie it to the much older diagnosis of Bell's acute exhaustive mania (DiMaio & DiMaio, 2006, p. 2). In 1849 Dr Luther Bell of the McClean Asylum for the Insane in Massachusetts published a paper in The American Journal of Insanity in which he described 40 cases of mania of sudden onset combined with the disorientation, confusion and fluctuating consciousness characteristic of delirium (Bell, 1849). Three quarters of these patients died but autopsy failed to reveal a convincing underlying cause. The delirium had been such a prominent feature of the cases described by Bell that his differential diagnosis had included delirium tremens, encephalitis, meningitis and advanced typhoid (perhaps making ‘Bell's mania’ somewhat of a misnomer). During the 171 years since Bell's paper, there have been sporadic reports of Bell's mania and of the very similar condition of lethal catatonia but the incidence of these two conditions has not been established (Fink, 1999; Mann et al., 1986, p. 1374).

The present-day proponents of excited delirium often invoke Bell's exhaustive mania as the cause of death in violently disturbed individuals who have been restrained, overlooking the fact that Bell's patients died three to four weeks after the onset of their severely agitated behaviour. By contrast the modern subjects whose death certificates record excited delirium tend to have been behaving dangerously only for a matter of hours before becoming unresponsive and dying, during or shortly after restraint. For example, here in the United Kingdom, the 1997 inquest into the death of the Gambian asylum seeker Ibrahima Sey, who died in custody after the police refused to allow his close friend to accompany him into Ilford police station, heard that 6 to 8 officers physically restrained him and used CS gas. Mr Sey died from restraint asphyxia but the psychiatric expert giving evidence on behalf of the Metropolitan Police, invoked Bells exhaustive mania as the cause of death. Counsel for Mr Sey's family challenged this attribution by pointing out that in marked contrast to Bells exhaustive mania cases, his mania had lasted only several hours.

This discrepancy does not trouble the proponents of excited delirium. In a 1999 paper, American forensic pathologists Steven Karch and Boyd Stephens credited Luther Bell with the first observation of excited delirium despite the ‘several hours’ of violent agitation prior to cardiac arrest in its victims today (Karch & Stephens, 1999, p. 110–111). They wrote: ‘…often, police come into contact with the deceased during the phase of psychotic agitation, *just as death is about to occur*’ (emphasis added) (Karch & Stephens, 1999, p. 111). Karch and Stephens described death in restrained stimulant drug abusers or agitated chronic psychotic patients as virtually inevitable. They too invoked Chan's physiological laboratory tests to rule out any specific mode of restraint, including hog-tying, as the cause of respiratory arrest, instead emphasising that death is very likely in ‘this generally fatal disease’ (Karch & Stephens, 1999, p. 112). For Steven Karch and Charles Wetli, excited delirium is itself deadly, regardless of whether or how the subject is restrained (Karch, Wetli, & Stratton, 1995).

It appears to us that the recasting of excited delirium as a lethal condition without an identifiable lesion allowed for the attenuation of the multi-factorial approach to death investigation that hitherto critically analysed each contributory step in the fatal sequence of events. Identifying excited delirium as the primary cause of a death in custody downgraded the causative role of other factors in the fatal cascade of events, such as the use of dangerous restraint. Despite academic papers warning of the dangers of restraint (O'Halloran & Lewman, 1993; Reay & Eisele, 1982; Reay, 1992), by the time of the DiMaios' influential work, excited delirium had been certified as the cause of many deaths in custody.

## The Braidwood enquiries

On 14 October 2007 at the Vancouver International Airport, an officer of the Royal Canadian Mounted Police (RCMP) used a CEW against Mr Robert Dziekanski, a Polish national, who, after being subdued and handcuffed, died within minutes. Following ‘immediate and intense’ public concern, the provincial government asked retired British Columbia supreme court judge Thomas Braidwood QC to head two commissions of enquiry: the first into police use of CEWs; the second into the circumstances of Mr Dziekanski's death. These enquiries, conducted in 2009–2010, represented a major turning point in the excited delirium story.

Before the enquiry sat, an article in the Canadian Medical Association Journal labelled Mr Dziekanski's death ‘A knee in the neck of excited delirium’ (Truscott, 2008). The author questioned two experts about the validity of excited delirium as a diagnosis. Dr Ian Dawe, director of psychiatric emergency services at St. Michael's Hospital in Toronto, described excited delirium as a ‘pop culture phenomenon’ and one that ‘doesn't have much currency among psychiatrists, although police, coroners and forensic pathologists use it.’ On the other hand, Dr Deborah Mash, professor of neurology at the University of Miami and a long-standing colleague of Charles Wetli, the co-inventor of excited delirium, claimed that ‘sudden death in the context of emotional stress is well known.’ She told the Journal she had evidence to suggest that excited delirium is a brain disease, arguing that it results from an interaction between a gene that remains silent until ‘triggered by something like alcohol, drugs, stress or sleep deprivation – anything that affects dopamine’.

Judge Braidwood's enquiry heard how the diagnosis of excited delirium was actively promoted by the leading manufacturer of CEWs. Dr Mike Webster, a consultant psychologist who had worked with police forces for 30 years, accused TASER International (now ‘Axon Enterprises Inc’) of ‘brainwashing’ police forces (Joyce, 2008). He told Judge Braidwood that Axon/TASER had conducted a ‘brilliant marketing scheme and created a lucrative business’ based on selling their weapons as a necessary tool when confronted with ‘excited delirium.’ He accused Axon/TASER of passing on the diagnosis of excited delirium to police forces, and he was critical of police for taking their information directly from the manufacturer.

Axon/TASER attends conferences for police chiefs and medical examiners, where it distributes literature on excited delirium, including free copies of the DiMaios' book. It has also sent unsolicited materials to a US medical examiner when an in-custody death occurred in his jurisdiction. According to a Reuters investigation, in August 2013 a Miami Dade police officer shot an 18-year-old with a CEW. The young man died an hour later. Within four hours of the death, the Miami-Dade Police Department received an email from TASER International. Marked ‘confidential’ and ‘timely and urgent’, it offered the police unsolicited advice on how to proceed, including advising Miami's medical examiner to send the dead teenager's brain tissue to Dr Deborah Mash. The email did not mention that Dr Mash had been paid $24 000 by TASER International to testify on its behalf in eight recent lawsuits against the company (Szep, Reid, & Eisler, 2017).

In 2002, Axon/TASER even released a statement for police forces to use if someone died in a CEW-related incident:

We regret the unfortunate loss of life. There are many cases where excited delirium caused by various mental disorders or medical conditions, that may or may not include drug use, can lead to a fatal conclusion (Garcia-Roberts, 2010).

Dr Webster told the Braidwood enquiry that as a result of Axon/TASER's efforts, ‘police and medical examiners are using the term [excited delirium] as a convenient excuse for what could be excessive use of force or inappropriate control techniques during an arrest’ (Balko, 2015).

In his first report Judge Braidwood found CEWs to pose a risk of serious injury or death. He made a series of recommendations regarding the appropriate use of such weapons. The definition of excited delirium that he included in his report's glossary reflected both the divergence of expert opinion and the term's very broad application:

Excited delirium – a controversial term used to describe a person who is highly agitated and often under the influence of stimulants/drugs or suffering from a mental condition. (Braidwood, 2009, p. 544).

Axon/TASER, a litigious company (Logan, 2013) with a long and successful record of using excited delirium to defend its weapons in court (Sullivan, 2007; Yeung et al., 2009), brought judicial review proceedings challenging Judge Braidwood's phase 1 report. It sought an order quashing all his findings as to the safety of its products; a declaration that he had failed to take relevant information into account; and an injunction restraining the judge from relying on his own research and findings as to the medical safety of these weapons when writing his phase 2 report on the circumstances of Mr Dziekanski's death. The Supreme Court dismissed the challenge (*Taser Int. v. British Columbia* (*Braidwood Study Commission*), 2010).

Judge Braidwood concluded that the cause of Mr Dziekanski's death will never be known with absolute certainty. He found that the most likely cause of death was Mr Dziekanski's hyperadrenergic reaction to the deployment of the CEW and physical altercation with the RCMP officers (Braidwood, 2010, p. 16). Vincent DiMaio's evidence that Mr Dziekanski was suffering from excited delirium was challenged by another forensic pathologist and ultimately not accepted by Judge Braidwood. Addressing the difficult task of identifying the cause of death in such cases, the judge warned that reliance on such terms may pre-empt investigation of the underlying organic cause and obscures the causative role of restraints.

## The ACEP task force

As British Columbia instructed Judge Braidwood to investigate the circumstances of Mr Dziekanski's death, south of the border the American College of Emergency Physicians (ACEP) contemporaneously established a ‘task force’ of experts to consider whether excited delirium is a real medical entity. Its panel, comprising mainly emergency physicians and forensic pathologists, included Drs Deborah Mash and Theodore Chan. Making no reference to Braidwood's enquiries, the task force concluded that excited delirium is ‘a real syndrome’ (ACEP Excited Delirium Task Force, 2009). In a celebratory article in Emergency Medicine News entitled ‘Identifying New Disease of Excited Delirium Syndrome Rejects Idea that Police Brutality Causes Deaths’, task force chair Dr Mark DeBard announced its ‘groundbreaking conclusion’ (DeBard, 2009). He declared ‘It is not often in a modern physician's career that a new disease, syndrome, or pathological process is described or recognised. It requires significant expert consensus to achieve this.’

The task force concluded that the new syndrome could be identified by a distinctive group of clinical and behavioural characteristics, a triad of delirium, psychomotor agitation and physiologic excitation, with between six and 10 separately identifiable characteristics. It occurs in stimulant abuse or serious mental illness.

A further panel of experts was established by the National Institute for Justice's Weapons and Protective Systems Technologies Center. (The NIJ is the arm of the US Department for Justice tasked with developing technology for use in law enforcement). Its members included Steven Karch, the DiMaios, Theodore Chan and eight other members of the ACEP task force. Reporting in 2011, the panel dismissed Braidwood's report as merely summarising the proffered expert testimony, arguing the judge made no effort to weigh the science for or against excited delirium (NIJ Weapons and Protective Systems Technologies Center, 2011, p. 3).

In 2011, members of the ACEP task force recommended the use of CEWs in cases of excited delirium to reduce the risk of acidosis from a prolonged physical struggle (Vilke, Bozeman, Dawes, DeMers, & Wilson, 2012*a*, p. 120). To reassure those critical of the use of CEWs in the restraint of individuals, a team funded by the US Department of Defence's Joint Non-Lethal Weapons Program investigated the biomedical impact of the use of CEWs on 31 police academy cadet volunteers (Kroll, Hail, Kroll, Wetli, & Criscione, 2018). The team included Charles Wetli and Mark Kroll, adjunct professor at the Department of Biomedical Engineering at the University of Minnesota in Minneapolis and a member of the corporate board of Axon/TASER. It concluded that CEW exposure did not elicit a stress response that could itself cause excited delirium through the release of serotonin (Kroll et al., 2018, pp. 481–482).

## A divergence in approaches in North America

This divergence between the US and Canadian approaches to excited delirium was accentuated by a restraint-related death in Halifax, Nova Scotia. Five months after Braidwood concluded his work, Judge Anne S Derrick lodged her report of her enquiry into the death in police custody of Howard Hyde on 22 November 2007. She rejected the claim that Mr Hyde died from excited delirium (Derrick, 2010, p. 184), expressed scepticism that it is a medical condition (Derrick, 2010, p. 186) and recommended its removal from Nova Scotia's policies and training for first responders (Derrick, 2010, p. 360).

Mr Hyde suffered from chronic schizophrenia characterised by paranoia, anxiety and agitation. Having been arrested for assault, he became terrified upon his arrival at the police station and a CEW was deployed when he struggled with officers. Following a further struggle he collapsed and stopped breathing. He was brought to hospital where his condition stabilised and he was returned to custody and later that day appeared in court. As it was too late in the day to arrange his release on bail, Mr Hyde was remanded in custody. Early the next morning, ahead of his return to court, he became fearful and attempted to get away from correctional officers. While Mr Hyde was restrained in his cell he stopped breathing. No pulse could be detected. He was pronounced dead in the hospital.

Dr Bowes, the Chief Medical Examiner of Nova Scotia, identified excited delirium as the cause of Mr Hyde's death. This conclusion was based on his consideration of the autopsy report, police reports, correctional officers' statements, Mr Hyde's medical records and video footage of the struggle. The forensic pathologist instructed to conduct the autopsy was not asked to provide his opinion as to the cause of death. However the expert evidence presented to the enquiry was sharply divided about excited delirium (Derrick, 2010, p. 181).

In rejecting excited delirium as the cause of Mr Hyde's death, Judge Derrick noted that many of its supposed features were absent in this case (Derrick, 2010, p. 184–185). She went through the list of symptoms that the American task force had demarcated as the pathognomonic features. Mr Hyde was not incoherent and had not failed to recognise or respond to the police. He was not impervious to pain as he screamed in agony when hit with the CEW. Whilst officers described him as being extremely strong, they were able to bring him down very rapidly. The suggestion that Mr Hyde was not ‘goal oriented’ was undermined by his intelligible responses to officers despite his paranoia and he remained solely focussed on escaping those who he felt were trying to hurt him – fears realised when the officers deployed the CEW. His body temperature was normal and only one officer thought to mention that he was sweating. Excited delirium, in terms of identifying a cause of death, was ‘a red herring’ (Derrick, 2010, p. 197).

Judge Derrick pointed out that ‘the use of excited delirium to explain sudden deaths with no anatomic findings implies that the person had something wrong with them that caused their inexplicable death’ (Derrick, 2010, p. 212). This formulation would allow such deaths to be classified as ‘natural’, which she felt was inappropriate in cases such as that of Mr Hyde.

## Greater scrutiny in Europe

Two reports published in 2017 cast further doubt on claims of deaths due to excited delirium. The use of restraint by police forces in the United Kingdom featured prominently in Dame Elish Angiolini's report on deaths in police custody. Her review was commissioned in 2015 by the then Home Secretary, Theresa May, after meeting with the families of Sean Rigg and Olaseni Lewis, both of whom died after being restrained by police officers. Sean Rigg was a 40-year-old Black musician who suffered from schizophrenia. He died on 21 August 2008 at Brixton police station, south London, following his arrest by Metropolitan Police Service officers. Olaseni Lewis was a 23-year-old Black graduate with plans for postgraduate study. He died on 4 September 2010 after being restrained by up to 11 police officers while he was seeking help as a vulnerable voluntary patient at the Bethlem Royal Hospital in Beckenham.

Angiolini highlighted that more than fifteen years since the death of Roger Sylvester, restraint-related deaths continue to occur, particularly in those suffering a mental health emergency (Angiolini, 2017, p. 32). She saw the emergence of the same themes in many of these deaths as indicative of a failure to learn lessons from the critical findings of previous inquests. Angiolini noted concerns that excited delirium is raised at inquests in ‘an attempt to explain away a death and deflect attention from the use of force’ (Angiolini, 2017, p. 39). She explained that reference to the term may result in it becoming the focus of an inquest, with the consequence that ‘the use of any restraint may be subsequently downplayed.’ Angiolini recommended that excited delirium never be used as a term that, by itself, can be identified as the cause of death (Angiolini, 2017, p. 44). She also called for the term to be removed from guidance to police officers. Given the extent of professional disagreement on excited delirium, she adopted the recommendation of one of the authors (Lipsedge, 2016) and urged pathologists, psychiatrists and practitioners of emergency medicine to collaborate in order to achieve consensus and clarity as to the medical understanding of restraint related deaths (Angiolini, 2017, p. 47).

The lack of consensus was made clear by a systematic review of the medical literature on excited delirium conducted by a team of emergency physicians at Lausanne University Hospital (Gonin, Beysard, Yersin, & Carron, 2017, p. 552). Whilst the review did not question the validity of excited delirium as a real clinical entity, it was highly critical of 66 published studies because of their limited levels of evidence. Although the ACEP task force had recommended that a diagnosis of excited delirium be based upon evidence of perceived abnormal behaviour and at least six of their 10 potential clinical criteria for such a diagnosis (Vilke et al., 2012*b*, p. 900), the Lausanne review team found many patients to have been diagnosed with excited delirium despite presenting with fewer than six of these diagnostic features. The most common features – including claims of superhuman strength, bizarre behaviour and unusual pain tolerance – did not appear with equal frequency and appeared not to be mandatory (Gonin et al., 2017, p. 561). The prevalence of excited delirium also appeared to ‘vary widely with context’. Whilst cases requiring out-of-hospital restraint were observed in fewer than two cases for 10 000 emergency calls for advanced life support, excited delirium was associated with more than 10% of deaths in police custody and said to represent more than 10% of CEW-related deaths.

## The absence of an agreed definition

The ACEP task force's 2009 White Paper cited an unreferenced ‘published observational study’ which suggested ‘the incidence of death among patients manifesting signs and symptoms consistent with ExDS is 8.3%.’ Strömmer et al. highlighted the problem with this assertion: the cited study – a 2007 publication by Barnett et al. (2007) – contained no mention of excited delirium, nor of a mortality rate from any cause (Strömmer, Leith, Zeegers, & Freeman, 2020, p. 685). The absence of a clear, consistent definition has made it difficult to estimate the incidence of excited delirium and has prevented a consensus emerging as to its mortality rate. On the one hand, there is the conviction that people presenting with these signs were always doomed, unable to alight from the ‘freight train of death’. This opinion is seen in Wetli, Mash and Karch's description of excited delirium as comprising ‘four components which appear in sequence: hyperthermia, delirium with agitation, respiratory arrest, and death’ (Wetli, Mash, & Karch, 1996). It is reflected in DiMaio and Dana's conclusion that deaths following the use of CEWs ‘appeared to be deaths due to excited delirium syndrome in individuals who coincidentally received Taser shocks’ (DiMaio & Dana, 2007, p. 170).

On the other hand, whilst the chair of the ACEP task force placed the mortality rate at a high 8–14% (DeBard, 2009, p. 3), a subsequent study by task force colleagues into police use of force in one Canadian city found that although 15% of forcibly arrested individuals had three or more concomitant signs of excited delirium, only one of 209 of these people actually died during or shortly after restraint (Hall et al., 2013, p. 102). Another study found that restraint related deaths in individuals said to be suffering from excited delirium declined by 33% between 1988 and 2011 (Michaud, 2016). The authors highlighted the ‘impact of warnings and recommendations from coroners’ inquest[s] on police officers’ training’ (Michaud, 2016, p. 34).

As the emergency physician Jared Strote commented, in the absence of a physiologic explanation ‘the use of the excited delirium diagnosis risks tautology: excited delirium (agitation with risk of sudden death) was present because death occurred after agitation’ (Strote, 2013). This point was made in evidence by Professor Anthony Brown, a senior emergency medicine specialist, at an inquest into a December 2009 custody death in Victoria, Australia. He took issue with another witness' statement that the deceased's presentation matched that of others said to have had excited delirium. He told the coroner:

I think that is just a description of what they were like before they died, and it doesn't help us predict who will die, why they have died and then, more importantly, how to prevent it. So it becomes an iterative sort of argument where it's almost like the cause of death because the circumstance and I think that's dangerous (Jamieson, 2015, p. 43).

A recent methodologically rigorous review confirmed that in the absence of aggressive restraint (eg manhandling, or the use of handcuffs in the prone position, or of hobble ties), severely agitated states are not inherently lethal (Strömmer et al., 2020, p. 680). It concluded that ‘the more likely it is that a death resulted from restraint, the more likely it is that the death will be attributed to [excited delirium], which allows for the restraint to be ignored as a cause’ (Strömmer et al., 2020, p. 684).

## The future for excited delirium

At first glance, there is reason to be pessimistic. In Canada, provincial court Judge Heather Lamoureux must not have read the Braidwood or Derrick reports when, in November 2011, she found that Gordon Bowe died an accidental death from excited delirium, rather than as a result of the CEW use and heavy-handed restraint by Calgary police officers. In the US, deaths that follow the use of restraint or a CEW continue to be chalked up to excited delirium. The use of the diagnosis of excited delirium to minimise the role of hazardous restraint has spread further afield, as shown by the medical reports of the deaths in custody of three men in Warsaw between 2013 and 2017 (Śliwicka, Szatner, & Borowska-Solonynko, 2019). An otherwise authoritative recent article on acute behavioural disturbance by Richard Stevenson and Derek Tracy minimises the risks of faulty restraint by asserting that ‘sudden death after the application of restraint is rarely due to the restraint procedure itself’ (Stevenson & Tracy, 2020).

However there is also reason for optimism. An editorial in the Globe and Mail newspaper responded to Judge Lamoureux's Public Fatality Inquiry report by saying Canada does not need a national debate over excited delirium, as that theory was put to rest by the Braidwood enquiries (‘Delirious Over Delirium’, 2012). Dismissing her suggestion for the creation of a nation-wide excited delirium database, the paper testily suggested that Canada's judges and policy makers should make the time to read Braidwood's reports. In Australia, in 2015, a coroner in Victoria concluded that excited delirium is neither appropriate nor helpful for ascribing the medical cause of a death (Jamieson, 2015, p. 52). (This despite a pathologist's report that identified excited delirium as the cause of death). In so doing the coroner referred to the scientific opposition to excited delirium, to the Braidwood reports and to Axon/TASER's activities to promote excited delirium, its financial relationship with many of those promulgating the diagnosis and its strategy of bringing legal proceedings against medical examiners in the US (Jamieson, 2015, p. 8).

Even in the United States, restraint-related deaths ascribed to excited delirium are being re-examined in the wake of George Floyd's murder. In a case of particular notoriety, the Governor of Colorado directed prosecutors to reopen their investigation of the death during police restraint of 23-year-old Elijah McClain in Aurora in August 2019 (BBC News, 2020). McClain died after three officers placed him in a chokehold and handcuffs and paramedics injected him with ketamine. The sedative may be administered in Colorado when someone is thought to be exhibiting ‘excited delirium’ (Nieberg, 2020). The officers later spoke of McClain's ‘incredible, crazy strength’ (the autopsy report showed McClain as 5 feet 6 inches tall and weighing only 140 pounds) (Tompkins, 2021). Despite there being no suggestion that McClain had broken any law, the initial investigation concluded with no criminal charges being brought against the officers. According to the Los Angeles Times, several police departments across the US are reconsidering how they train officers on the use of force (Zou, Wailoo, & O'Toole, 2020). The Los Angeles Police Department told the newspaper it will not be hosting any further such training by a Minnesota company called the Force Science Institute, which counts two ACEP task force members amongst its trainers. The Institute's guidance has been criticised for being ineffective, for fostering fear amongst officers that can lead to unnecessary force and for the promulgation of excited delirium.

The American Psychiatric Association Board of Trustees adopted a position statement in December 2020 which declared that excited delirium should not be used as a diagnosis because it is non-specific and lacks clear diagnostic criteria (American Psychiatric Association, 2020). The statement noted that excited delirium ‘had been invoked to explain or justify injury or death to individuals in police custody’ and that the term is disproportionately applied to black men in police custody.

Here in England and Wales, Dr Meng Aw Yong, medical director for Forensic Healthcare Services of the Metropolitan Police, lectures on the dangers of acute behavioural disturbance, not excited delirium, and says ABD is ‘itself not a definitive condition’ but rather a description of ‘a spectrum of behaviours, signs and symptoms’ (Aw-Yong, 2020, p. 228). Dr Aw Yong is seeking to persuade ambulance services to upgrade these cases to matters of the highest priority. He emphasises the dangers of excessively long restraint and has made it his mission to ensure there are national guidelines for responding to people presenting with ABD. Such an approach takes us further from the theory of excited delirium as an intrinsically fatal condition and emphasises the medical risks associated with restraint. We applaud this balanced view of those emergency situations where verbal de-escalation has failed and where paramedics, law enforcement personnel, nurses and medical staff have no alternative but to resort to physical restraint for the protection of the agitated person and of other people.

## Conclusion

The term excited delirium was filched nearly 40 years ago from the standard medical lexicon in order to explain the sudden deaths in police custody of young men whose wild and alarming behaviour had led to their arrest. Despite the absence of specific findings at autopsy and warnings that police use of neck and choke holds were medically risky and potentially lethal, medical examiners began to identify excited delirium as the principal cause of death. This diagnosis has been invoked at enquiries and inquests to exculpate officers whose dangerous use of restraint may have significantly contributed to the death of these publicly distressed individuals.

Death investigations have rarely been in a position to ask of excited delirium the important questions that the sociologist Phil Brown urges us to ask of any new phenomenon or diagnosis, namely: ‘Why did the condition get identified at a certain point in time?’, and ‘Who benefits, or at least avoids trouble, by its identification and action?’ (Brown, 1995). We have striven to show how police officers and the manufacturers of CEWs and restraint devices have relied upon the diagnosis of excited delirium to prevent scrutiny and limit liability. As Strote argued, ‘…if all agitated patients who die unexpectedly during restraint can be easily dismissed as suffering from Excited Delirium Syndrome, excessive use of force may be overlooked or excused’ (Strote, 2013).

The killing of George Floyd brought renewed scrutiny of police restraint techniques and use of CEWs. We predict that a diagnosis of excited delirium as the principal cause of a restraint-related death will continue to be discredited due to its lack of validity, specificity and predictive utility (not to mention the absence of a pathognomonic lesion at autopsy). We welcome the new preference for the descriptive term acute behavioural disturbance as a clinically valid picture of the highly agitated young men whose urgent need for emergency care poses such a challenge to first responders.

However we recognise the risk that the term acute behavioural disturbance might be misused at enquiries or inquests as a proxy for excited delirium, to downplay the role of faulty and negligent restraint. We are concerned that at the recent inquest into the death of Kevin Clarke, a 35 year old Black man who was experiencing a severe mental health crisis in a public place, the jury recorded the primary medical cause of death as ‘acute behavioural disturbance (in a relapse of schizophrenia) leading to exhaustion and cardiac arrest, contributed to by restraint struggle and being walked’ (Harris, 2021). We would tentatively suggest ABD's replacement with an adjectival description, such as ‘severely agitated person in distress’ (‘SAPID’ could be used as an emergency services call sign). Like excited delirium before it, the misappropriation of the term acute behavioural disturbance must not be allowed to downplay the threat that faulty restraint poses to life in these highly challenging situations.
